# Interaction between physical activity and outdoor time on allostatic load in Chinese college students

**DOI:** 10.1186/s12889-022-12518-0

**Published:** 2022-01-27

**Authors:** Dan Zhang, Tingting Li, Yang Xie, Shuman Tao, Yajuan Yang, Liwei Zou, Yang Qu, Shuang Zhai, Fangbiao Tao, Xiaoyan Wu

**Affiliations:** 1 Department of Maternal, Child and Adolescent Health, School of Public Health, Anhui Medical University, Hefei, China; 2 MOE Key Laboratory of Population Health Across Life Cycle, Hefei, China; 3 NHC Key Laboratory of Study on Abnormal Gametes and Reproductive Tract, Hefei, China; 4 Anhui Provincial Key Laboratory of Population Health and Aristogenics, Anhui Medical University, Hefei, China; 5 The Second Hospital of Anhui Medical University, Hefei, China; 6 School of Nursing, Anhui Medical University, Hefei, China

**Keywords:** Allostatic load, Physical activity, Outdoor time, College student

## Abstract

**Background:**

Physical activity (PA) deficiency, outdoor time reduction during college have been associated with higher cumulative physiological burden as measured by allostatic load (AL). Therefore, the present research sought to analyze the independent and interaction effects of PA and outdoor time on AL in college students.

**Methods:**

A cross-sectional survey was conducted in two universities from April to May 2019. Self-assessment questionnaire and International Physical Activity Questionnaire Short Version (IPAQ-SF) were used in the investigation, AL level was assessed according to the results of biochemical examination, blood pressure and human body morphological measurements. Binary Logistic Analysis was used to analyze the relationships between PA, outdoor time and AL.

**Results:**

The prevalence of low PA, low outdoor time and high AL were 16.3%, 71.1% and 47.6%, respectively. Low PA (*OR*=1.83, 95%*CI*: 1.20~2.78) and low outdoor time (*OR*=1.90, 95%*CI*: 1.35~2.67) are independently associated with high AL (*P*<0.05, for each). Interaction analysis indicated that low PA and low outdoor time were interactively associated with high AL (*OR*=2.93, 95%*CI*: 1.73~4.94, *P*<0.05).

**Conclusions:**

There were the significant independent and interaction effects between PA and outdoor time on AL. In the future, college students’ physical education can be arranged reasonably to reduce the health risks.

## Introduction

The concept of AL was introduced by McEwen and Stellar in 1993 [1] and it offers a comprehensive index for understanding how exposure to environmental stressors during development can lead to a result of multi-system maladjustment and poor health. As a comprehensive index [1], AL involves multi-system including the sympathetic adrenomedullary system (SAM), the hypothalamic pituitary adrenal (HPA) axis, indices of inflammation, immune functioning, lipid metabolism and it’s important to use clinimetric approach for a more comprehensive assessment of allostatic load and overload. Therefore, AL represents the cumulative effect of daily life experiences involving both ordinary events (long-standing life situations) and major challenges (life events), including the physiological consequences of resulting health damaging behaviors. Accordingly, when environmental challenges exceed the individual ability to cope, then allostatic overload will occur [1].

Most college students range in age from approximately 18 to 22 years, and college is therefore a transitional period from adolescence to adulthood. This age-span is a critical period for the development of healthy lifestyles and behaviors that are important throughout the lifespan [2]. However, under the double burden of study and life, the life style of college students has changed, which is characterized by less physical activities (PA), especially outdoor activities [2]. According to the latest World Health Organization (WHO) survey, about 80 per cent of adolescents worldwide do not meet the recommended level of PA per day [3].

In addition, numerous studies have shown that less PA is one of the important risk factors for health, less PA is associated with cardiovascular disease, type 2 diabetes, colon cancer, and other important diseases [4, 5]. The health consequences of PA and outdoor time involve multiple physiological systems. Therefore, as a comprehensive index, AL can be used to measure the multi-system influence of PA and outdoor time on organism. Through the comprehensive index AL, we can understand that how PA and outdoor time gets under the skin to influence biological processes that are highly relevant to physical health and mental well-being.

While PA and outdoor time has been linked in several studies to metabolic, inflammation and cardiovascular risk among adolescent [6–8], no previous research directly examined the independent and interactional influences of PA and outdoor time on cumulative multisystem dysregulation among college studentsadolescent. Therefore, the present research first sought to analyze the independent effects of PA and outdoor time on AL in Chinese college students, and then sought to explore the interaction effects between PA and outdoor time on AL.

## Material and method

### Study participants

The subjects were from the College Student Behavior and Health Cohort Study, which is an ongoing cohort designed to focue on behaviors, physical and mental health in college students from the first year to the third year in China. Baseline data was analyzed in the present study. Firstly, between April to May 2019, two cities were selected by convenient sampling from Hefei, Anhui Province, and Shangrao, Jiangxi Province. Then, a medical university and a comprehensive normal university were selected based on stratified cluster sampling. We randomly selected two colleges from each university and surveyed all first-year students in each college. The baseline survey included electronic questionnaires scanned with smartphones and physical examination. We received a total of 1 135 valid questionnaires that collected baseline information on sociodemographic and health-related factors, and the response rate was 98.6%. In addition, 778 subjects underwent physical examinations, including biochemical examinations, blood pressure and human body morphological measurements.

The current study was approved by the Ethics Committee of Anhui Medical University (No. 20170291). Written informed consent was obtained from all of the participants. Free medical examination is available to all participants. The study excluded people who were taking medication or undergoing psychotherapy, the final sample was 729 college students.

### Sociodemographic Data

Sociodemographic data for participants were collected by questionnaire, including age, gender (male or female), registered residential background (rural or urban), only child, parents’ education level (less than high school degree or more), self-reported family economy (bad, general or good).

### Allostatic load

The subjects of this study were sent to the local third-class hospitals for medical examination, measurement of height and weight using a fully automatic electronic height and weight meter. Height was measured to the nearest 0.1 cm and weight was measured to the nearest 0.1 kg. BMI was calculated as weight in kilograms divided by the square of height in meters. Waist measurement is accurate to 0.1 cm. The subjects had to rest for a few minutes before their blood pressure was measured, the resting blood pressure was then measured using an electronic sphygmomanometer. The subjects were given 5 ml of fasting venous blood, then lipid/lipoprotein (cholesterol, high-density lipoprotein, low-density lipoprotein, triglyceride), fasting plasma glucose, insulin, and high sensitivity c-reactive protein were measured.

A total of 11 biomarkers from three types of biological systems were used to assess AL level of college students: Cardiovascular system (cholesterol, high-density lipoprotein, low-density lipoprotein, triglyceride, systolic and diastolic pressure); Metabolic system (waist circumference, BMI, insulin and fasting blood glucose); Immune system (high-sensitivity C-reactive protein). The blood pressure threshold was defined by the screening for elevated blood pressure among children and adolescents aged 7~18 years (WS/T 610-2018) [9] Waist circumference was defined by the high waist circumference screening threshold among children and adolescents aged 7~18 years (WS/T 611-2018) [10]. The threshold of BMI was defined by the screening for overweight and obesity among school-age children and adolescents (WS/T 586-2018) [11]. The other biomarkers refer to the demarcation standard of AL index of college students by Currie CL et [12]. The specific threshold criteria are shown in Table 1.

**Table 1:** Threshold criteria for AL biomarkers (*N*=729)

Variables	***Means*** ± ***SD***	Threshold criteria for AL biomarkers	
		Males	Females
Cardiovascular			
Cholesterol (mmol/L)	4.08 ± 0.69	≥6.10	≥6.20
High-density lipoprotein (mmol/L)	1.44 ± 0.27	≤1.22	≤1.45
Low-density lipoprotein (mmol/L)	2.08 ± 0.50	>2.76	>2.82
Triglyceride (mmol/L)	0.89 ± 0.44	>2.16	>1.67
Systolic pressure (mm Hg)	115.13 ± 12.89	>140	>140
Diastolic pressure (mm Hg)	70.93 ± 9.08	>90	>90
Metabolic			
Waist circumference (cm)	71.05 ± 7.80	>83.0	>76.1
BMI (kg/m^2^)	20.71 ± 2.57	>23.8	>23.9
Insulin (uIU/mL)	5.46 ± 4.56	>20	>20
Fasting blood glucose (mmol/L)	4.55 ± 0.41	>5.8	>5.5
Immune			
High-sensitivity C-reactive protein (mg/L)	1.12 ± 2.02	≥8	≥8
Total AL score	1.59 ± 1.16		

*BMI* Body Mass Index, *AL* Allostatic load, *SD* Standard Deviation

Each biomarker is defined a value according to the threshold criteria, biomarkers above the threshold (high-density lipoprotein below the threshold) are defined a score of 1. The total AL score was obtained by sum of 11 biomarkers, and the range of score was 0~11. The higher the score, the more serious the physiological systems dysregulations was. In this study, AL score<2 was defined as low level and ≥2 as high level [13].

### Physical activity

The International Physical Activity Questionnaire Short Version (IPAQ-SF) was used to measure PA of college students in the past week [14, 15]. PA level was divided into low physical activity (LPA = 3.3 metabolicequivalent [METs]), moderate physical activity (MPA = 4.0 METs) and above.

*A certain intensity of physical activity level = the activity corresponding to the Met assigned value × weekly frequency (d) × daily time (min)*

The criteria for low physical activity level are no physical activity reported or energy expenditure not enough to the MPA criteria. The moderate physical activity and above level is any combination of activities of three intensity ranges of at least EE ≥600 MET min/week.

### Outdoor time

Two questions from the Young Risk Behavior Surveillance System questionnaire were modified to measure outdoor time [16]. Weekday outdoor time was measured by“In the last 4 weeks, the average daily daytime outdoor time was ______minutes on weekdays?”. Free day outdoor time was measured by “In the last 4 weeks, the average daily daytime outdoor time on the head without cover was ______minutes on free days?”

$$Total\;outdoor\;time\;=\;(outdoor\;time\;on\;weekdays\;\times\;5\;+\;outdoor\;time\;on\;free\;days\;\times\;2)/7$$

Referring to the Action for a healthy China (2019) [17], the study subjects were divided into two groups with 2h as the cut-off value, ≥2h as the high level, <2h as the low level.

### Covariates

Health behaviors included cigarette use, alcohol use, quality of sleep and symptoms of depression. Two questions from the Young Risk Behavior Surveillance System questionnaire were modified to measure current cigarette and alcohol use. Cigarette use was measured by “How many days did you smoke during the past month?” Alcohol use was measured by “How many days did you have at least one drink during the past month?” The answers are recoded into “yes” or “no”. Quality of sleep was assessed by the Pittsburgh Sleep Quality Index (PSQI).

### Statistical analysis

Statistical analysis was performed using SPSS version 23.0 (Statistical Package for the Social Sciences). The Chi-square test was performed to compare the incidence of AL level among different sociodemographic variables, PA, and outdoor time. Binary logistic analysis was used to analyze the relationships between PA, outdoor time and AL. Odds ratios (ORs) and 95% confidence intervals (95%CIs) were calculated for the explanatory factors and adjusted for confounding factors, including quality of sleep, sex, smoking and self-reported family economy. Statistical significance was set at *P*<0.05.

## Results

### Characteristics of participants

Table 2 depicts the sample characteristics. There were responses from 729 college students aged between 16 and 22 years old (*mean* ± *SD*: 19.0±3.07 years), 237 were males (32.5%) and 492 were females (67.5%). The high level of AL was detected in 47.6% (347/729) of college students, female students’ AL level higher than males (*P*<0.05). College students with higher economic status were more likely to experience higher level of AL (*P*<0.05). Compared with smokers, non-smoking college students showed higher levels of AL (*P*<0.05).

**Table 2:** Distribution of demographics characteristics of college students (*N*=729)

Variables	AL level		***χ***^**2**^	***P*** value
	Low (***n***=382)	High (***n***=347)		
Gender			14.21	<0.001
Males	148 (62.4)	89 (37.6)		
Females	234 (47.6)	258 (52.4)		
Registered residence			0.41	0.524
Rural	209 (51.4)	198 (48.6)		
Urban	173 (53.7)	149 (46.3)		
Only child			0.18	0.670
Yes	96 (51.1)	92 (48.9)		
No	286 (52.9)	255 (47.1)		
Paternal education			0.24	0.622
<12 years	338 (52.1)	311 (47.9)		
≥12 years	44 (55.0)	36 (45.0)		
Maternal education			0.40	0.526
<12 years	358 (52.1)	329 (47.9)		
≥12 years	24 (57.1)	18 (42.9)		
Household economic status			8.77	0.012
High	12 (30.0)	28 (70.0)		
Moderate	289 (54.2)	244 (45.8)		
Low	81 (51.9)	75 (48.1)		
Cigarette use			18.83	<0.001
Yes	39 (83.0)	8 (17.0)		
No	343 (50.3)	339 (49.7)		
Alcohol use			3.82	0.051
Yes	92 (59.4)	63 (40.6)		
No	290 (50.5)	284 (49.5)		
Quality of sleep			0.027	0.871
High	334 (52.5)	302 (47.5)		
Low	48 (51.6)	45 (48.4)		

The figures in () are composition ratio or detection rate/%

*AL* Allostatic load

### The Distribution Characteristics of PA and Outdoor Time

Table 3 depicts the distribution characteristics of PA and outdoor time. College students who were low PA reported higher rates of AL (58.8% VS 45.4%, respectively, *P*=0.007). Higher rates of AL were also observed in those with low level of outdoor time (52.9% VS 34.6%, respectively, *P*<0.001). Further analysis of work days and free days shows that college students who were low free day outdoor time (51.0% VS 38.4%, respectively, *P*=0.002) or low work day outdoor time (52.9% VS 28.8%, respectively, *P*<0.001) reported higher rates of AL.

**Table 3:** Detection rate of AL level in college students of different outdoor time and PA

Variables	AL level		***χ***^**2**^	***P*** value
	Low (***n***=382)	High (***n***=347)		
Total outdoor time			20.13	<0.001
High	138 (65.4)	73 (34.6)		
Low	244 (47.1)	274 (52.9)		
Free day outdoor time			9.26	0.002
High	122 (61.6)	76 (38.4)		
Low	260 (49.0)	271 (51.0)		
Work day outdoor time			29.20	<0.001
High	114 (71.3)	46 (28.8)		
Low	268 (47.1)	301 (52.9)		
PA			7.18	0.007
Moderate and above	333 (54.6)	277 (45.4)		
Low	49 (41.2)	70 (58.8)		

*AL* allostatic load, *PA* physical activity

### Associations of PA, outdoor time and AL

**Fig. 1:**
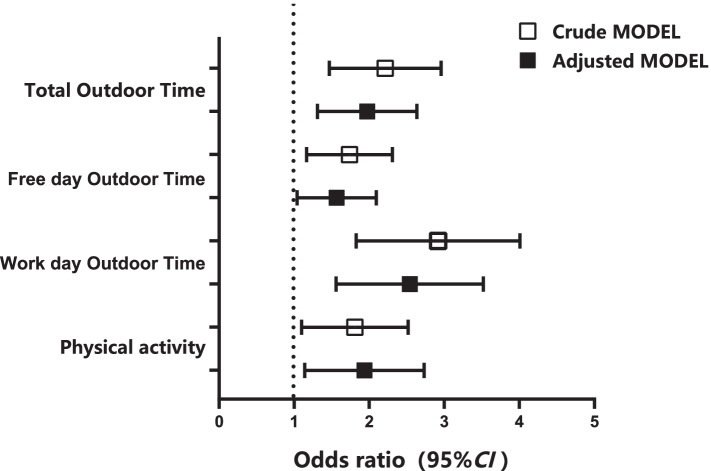
Associations of Physical activity, outdoor time and Allostatic Load

After adjusting for confounding factors including sex, smoking and self-reported family economy, results from binomial logistic regression analysis showed that low PA (*OR*=1.83, 95%*CI*: 1.20~2.78) and low outdoor time (*OR*=1.90, 95%*CI*: 1.35~2.67) are independently associated with high AL (*P*<0.05, for each, Table 4). Then, we stratified the data based on work day, free day, the results showed that both work day outdoor time (*OR*=2.41, 95%*CI*: 1.63~3.58) and free day outdoor time (*OR*=1.51, 95%*CI*: 1.07~2.12) are independently positively associated with AL (*P*<0.05, for each, Table 4, Fig. 1).

**Table 4:** Logistic regression analysis of outdoor time, PA level and AL of college students

Variables	Crude MODEL		Adjusted MODEL ^a^	
	*OR* (95%*CI*)	*P* value	*OR* (95%*CI*)	*P* value
Total outdoor time				
High	1		1	
Low	2.12 (1.52~3.00)	<0.001	1.90 (1.35~2.67)	0.001
Free day outdoor time				
High	1		1	
Low	1.67 (1.20~2.34)	0.002	1.51 (1.07~2.12)	0.019
Work day outdoor time				
High	1		1	
Low	2.78 (1.90~4.07)	<0.001	2.41 (1.63~3.58)	<0.001
PA				
Moderate and above	1		1	
Low	1.72 (1.15~2.56)	0.008	1.83 (1.20~2.78)	0.005

*AL* allostatic load, *PA* physical activity, *95%CI* 95% confidence interval, *OR* odds ratio

^a^ Adjusted MODEL controlled quality of sleep, household finances, gender and smoking

### Interactions of PA, and outdoor time with AL

The results of regression analyses examining the interactions of PA and outdoor time with AL were shown in Table 5. Table 5 shows crude and adjusted *OR* (95%*CI*) for AL in those with low PA or low outdoor time, low PA or high outdoor time, high PA or low outdoor time compared with the reference group (high PA or high outdoor time). There was a positive interaction effects between PA and outdoor time on AL (*P*<0.05), low PA college student with low outdoor time were more likely to be with high AL (*OR*=3.07, 95%*CI*: 1.86~5.07). After adjusting for confounding factors, the positive interaction effects remained significant (*OR*=2.93, 95%*CI*: 1.73~4.94, *P*<0.05) (Table 5).

**Table 5:** Interactions of PA, Outdoor time and AL in College Students

Outdoor time×PA	Crude MODEL			Adjusted MODEL ^a^		
	*B*	*OR* (95%*CI)*	*P* value	*B*	*OR* (95%*CI)*	*P* value
High×Moderate and above	1			1		
High×Low	0.93	2.54 (1.00~6.46)	0.050	1.10	3.01 (1.14~7.99)	0.027
Low×Moderate and above	0.79	2.19 (1.53~3.14)	<0.001	0.67	1.96 (1.14~2.84)	0.001
Low×Low	1.12	3.07 (1.86~5.07)	<0.001	1.07	2.93 (1.73~4.94)	<0.001

*AL* allostatic load, *PA* physical activity, *95%CI* 95% confidence interval, *OR* odds ratio

^a^ Adjusted MODEL controlled quality of sleep, household finances, gender and smoking

## Discussion

This study assessed, for the first time, the independent and interaction effects between PA and outdoor time on AL, a measure of biological multi-system dysregulation among a sample of 729 Chinese college students. The prevalence of low PA, low outdoor time and high AL were 16.3%, 71.1% and 47.6%, respectively. Participants with low PA and outdoor time exhibit higher AL.

We observed that 16.3% and 71.1% of the participants had low PA and low outdoor time, which was similar to Brazilian adolescents (PA: 16.2%) [18] and slightly lower than Chinese rural adolescents (outdoor time: 82.0%) [19]. PA deficiency and outdoor time reduction of college students is a common phenomenon, which has become a public health problem all over the world, which may be related to heavy academic load and too much sedentary time of college students.

We investigated high AL in 47.6% of participants, which was the same as Calcaterra V’s investigation (53.6%) [20]. However, another study in the United States [21] showed that the detection rate of high level (score≥2) of AL among college students was 79%, which was higher than our study. More than half of college students are at Al high level, which may be due to insufficient physical activity and lack of outdoor time [22, 23].

### Independent effects

Previous studies have also explored the relationship between PA and outdoor time and AL. Forrester SN et al. reported that moderate to high PA was beneficial for lower AL [22]. Findings from the Generation XXI birth cohort revealed that negative association between outdoor time and AL in adolescence, suggesting that the exposure of green environment may contribute to improve adolescent health [23]. D’Alessio et al. also discusses the beneficial effects of yoga as a kind of PAs on physical health and the reduction of AL [24]. Thus, it can be inferred that low PA and low outdoor time exposure can lead to elevated levels of AL in college students, leading to increased health risks.

In addition, in our study, we didn’t find a difference in the relationship between free day outdoor time, work day outdoor time, and AL levels. We not only observed a positive association between short free day outdoor time and high AL, but also observed a positive association between work day outdoor time and Al. It’s the opposite of a study of rural Chinese teenagers [25], work day outdoor time was associated with cardiovascular and metabolic health, while free day outdoor time was not found to be associated. It possibly because college students have more freedom to schedule outdoor time on work days and free days.

### Interaction effects

Although there have been a few studies have illuminated significant independent effects between PA and outdoor time on AL. Yet study on the interaction effects of PA and outdoor time on AL is lacking. In fact, increasing outdoor time has been shown to be an important strategy for increasing PA levels, increasing the outdoor time and the level of PA the same time have a more obvious impact on the health of the body [4]. A systematic review by Thompson Cocoon J et al. suggests that the health benefits of PA outdoors are greater than those of PA indoors [26].

The review from Gorman S et al. indicated that outdoor time could synergise with physical activity to prevent metabolic dysfunction, particularly that related to lifestyle diseases of obesity, type 2 diabetes and metabolic syndrome [27]. A series of new studies have shown that outdoor physical activity has varying degrees of impact on metabolism, cardiovascular and immune systems [28, 29], which can be used as indirect evidence to support the effect of PA and outdoor time interaction on AL. In the meantime, our study provides further evidence of the positive interaction effect between low PA and low outdoor time on AL and produce the different degree influence to the organism each physiological system.

### Biological mechanism

AL reflects the cumulative effect of stress in daily life, as a multi-system comprehensive index, it can be used as an early predictor of poor health or function. Our study found a correlation between AL and PA and outdoor time, which were generally consistent with emerging evidence from research on physical fitness development of adolescent [29], research on PA outdoor of college students suggesting that low level of PA and outdoor time could have an adverse effect on physical health and appeared to be associated with increased activation in the nervous, cardiometabolic and immune systems.

The present research adds to a growing base of evidence that suggests positive PA and outdoor time act as mechanisms that modify biological pathways associated with health risk. Chung WK et al. reported that low level PA outdoor adolescents be associated with higher BMI among a prospective study of the Netherlands [30]. A recent intervention study from Wuhan, China, revealed that PA outdoor improves the metabolic profile, cardiorespiratory fitness and insulin sensitivity in university students [31]. Contrepois K et al. conducted a study on the biological effects of PA on cardiovascular, metabolic and immune pathways. The results showed that, PA is associated with inflammatory response [32]. Less PA induced an undesirable inflammatory response with increased transcripts of ‘B cell receptor’, ‘NF-kB signaling’ and many interleukin signaling pathways. They also detected many pathways associated with cardiovascular related signaling highlighting the correlation between PA and cardiovascular health. As our research has shown that low PA and outdoor time exposure can affect physiological processes in college students.

### Research Prospect

Studies are now increasingly reporting on allostatic load in younger populations, including children and adolescents [12]. However, elderly people have higher AL levels than other groups due to their lack of physical activity and outdoor time in old age, as well as long-term experience of various chronic stresses. Therefore, in the future, researchers can further explore the impact of PA and outdoor time on other populations, especially the elderly AL.

### Limitations and Strengths

Some aspects of the study constitute possible limitations. First, the cross-sectional analysis precludes causal interpretation, and further longitudinal studies are needed to establish a causal relationship between PA and outdoor time and Al. Second, there are confounding factors including neighborhood quality, whichcontribute to AL that may not have been measured and included in analyses. Third, this study used self-report to evaluate the PA and outdoor time, so it may not be able to avoid reporting bias; however, the large sample size available for analysis reduces the likelihood of this having a meaningful impact on the results presented here. At last, the study objects were all college students, which have their own group characteristics, so the study may have selection bias, the results of the study to the general population extrapolation may be limited. However, these data were randomly selected which allows control for socioeconomic confounders and provides strong support for an association between PA, outdoor time and AL. Despite these limitations, this study makes several contributions that could have important implications. Our study uses AL as a comprehensive index to objectively and quantitatively evaluate the biological functions of multiple systems, which is superior to single-dimensional biomarkers. This study also addresses a gap in the literature by examining the independent and interaction effects between PA and outdoor time on AL in college student. Last but not least, we focus on the health risks of college students and emphasize the impact of early risk exposure on the whole life cycle.

## Conclusions

In our study, PA and outdoor time are cross-sectional associated with AL, with interactions of PA and outdoor time on AL. This research extends previous findings on the detrimental effects of PA and outdoor time by exploring and proposing a biological mechanism by which it exert effects on physical health. In the future, college students’ physical education can be arranged reasonably, and outdoor activities can be advocated to reduce the health risks of college students.

## Data Availability

The datasets generated and analysed during the current study are not publicly available because the author does not have permission to share the data but are available from the corresponding author on reasonable request.

## References

[1] McEwen BS, Stellar E (1993). Stress and the individual. Mechanisms leading to disease. Arch Intern Med.

[2] Hutchesson MJ, Duncan MJ, Oftedal S, Ashton LM, Oldmeadow C, Kay-Lambkin F, Whatnall MC (2021). Latent class analysis of multiple health risk behaviors among Australian university students and associations with psychological distress. Nutrients..

[3] Guthold R, Stevens GA, Riley LM, Bull FC (2020). Global trends in insufficient physical activity among adolescents: a pooled analysis of 298 population-based surveys with 1·6 million participants. Lancet Child Adolesc Health.

[4] Gray C, Gibbons R, Larouche R, Sandseter EB, Bienenstock A, Brussoni M, Chabot G, Herrington S, Janssen I, Pickett W, Power M, Stanger N, Sampson M, Tremblay MS (2015). What is the relationship between outdoor time and physical activity, sedentary behaviour, and physical fitness in Children. A systematic review. Int J Environ Res Public Health.

[5] Aune D, Schlesinger S, Leitzmann MF, Tonstad S, Norat T, Riboli E, Vatten LJ (2021). Physical activity and the risk of heart failure: a systematic review and dose-response meta-analysis of prospective studies. Eur J Epidemiol.

[6] Harada K, Lee S, Lee S, Bae S, Harada K, Suzuki T, Shimada H (2017). Objectively-measured outdoor time and physical and psychological function among older adults. Geriatr Gerontol Int.

[7] Gorman S, Larcombe AN, Christian HE (2021). Exposomes and metabolic health through a physical activity lens: a narrative review. J Endocrinol.

[8] Chen ST, Stevinson C, Yang CH, Sun WJ, Chen LJ, Ku PW (2021). Cross-sectional and longitudinal associations of outdoor walking with overall mental health in later life. Exp Gerontol.

[9] National Health and Family Planning Commission. Screening for elevated blood pressure among children and adolescents aged 7~18 years, 2018. http://www.nhc.gov.cn/wjw/pqt/201807/6cee88c1d050493ab50a411a2978f901.shtml

[10] National Health and Family Planning Commission. Screening for overweight and obesity among school-age children and adolescents, 2018. http://www.nhc.gov.cn/wjw/pqt/201807/417de6982ab8493b91aba925b51a8a19.shtml

[11] National Health and Family Planning Commission. Screening for overweight and obesity among school-age children and adolescents, 2018. http://www.chinanutri.cn/fgbz/fgbzhybz/201804/t20180418_162494.html

[12] Currie CL, Motz T, Copeland JL (2020). The impact of racially motivated housing discrimination on allostatic load among Indigenous university students. J Urban Health.

[13] Theall KP, Drury SS, Shirtcliff EA (2012). Cumulative neighborhood risk of psychosocial stress and allostatic load in adolescents. Am J Epidemiol.

[14] Ács P, Veress R, Rocha P, Dóczi T, Raposa BL, Baumann P, Ostojic S, Pérmusz V, Makai A (2021). Criterion validity and reliability of the International Physical Activity Questionnaire-Hungarian short form against the RM42 accelerometer. BMC Public Health.

[15] Nguyen LTK, Do BN, Vu DN, Pham KM, Vu MT, Nguyen HC, Tran TV, Le HP, Nguyen TTP, Nguyen QM, Tran CQ, Nguyen KT, Yang SH, Chao JC, Van Duong T (2021). Physical activity and diet quality modify the association between comorbidity and disability among stroke patients. Nutrients..

[16] Bull FC, Al-Ansari SS, Biddle S, Borodulin K, Buman MP, Cardon G, Carty C, Chaput JP, Chastin S, Chou R, Dempsey PC, DiPietro L, Ekelund U, Firth J, Friedenreich CM, Garcia L, Gichu M, Jago R, Katzmarzyk PT, Lambert E, Leitzmann M, Milton K, Ortega FB, Ranasinghe C, Stamatakis E, Tiedemann A, Troiano RP, van der Ploeg HP, Wari V, Willumsen JF (2020). World Health Organization 2020 guidelines on physical activity and sedentary behaviour. Br J Sports Med.

[17] National Board of Health, Healthy China Initiative (2019~2030)[ EB/OL ]. [2020-03-09]. http://www.gov.cn/xinwen/2019-07/15/content_5409694.htm

[18] Cheng LA, Mendonça G, Farias Júnior JC (2014). Physical activity in adolescents: analysis of the social influence of parents and friends. J Pediatr.

[19] Zhao Y, Guo Y, Xiao Y, Zhu R, Sun W, Huang W, Liang D, Tang L, Zhang F, Zhu D, Wu JL (2020). The Effects of Online Homeschooling on Children, Parents, and Teachers of Grades 1-9 During the COVID-19 Pandemic. Med Sci Monit.

[20] Calcaterra V, Vinci F, Casari G, Pelizzo G, de Silvestri A, De Amici M, Albertini R, Regalbuto C, Montalbano C, Larizza D, Cena H (2019). Evaluation of allostatic load as a marker of chronic stress in children and the importance of excess weight. Front Pediatr.

[21] Currie CL, Copeland JL, Metz GA (2019). Childhood racial discrimination and adult allostatic load: The role of Indigenous cultural continuity in allostatic resiliency. Soc Sci Med.

[22] Forrester SN, Leoutsakos JM, Gallo JJ, Thorpe RJ, Seeman TE (2019). Association between allostatic load and health behaviours: a latent class approach. J Epidemiol Community Health.

[23] Ribeiro AI, Tavares C, Guttentag A, Barros H (2019). Association between neighbourhood green space and biological markers in school-aged children. Findings from the Generation XXI birth cohort. Environ Int.

[24] D'Alessio L, Korman GP, Sarudiansky M, Guelman LR, Scévola L, Pastore A, Obregón A, Roldán EJA (2020). Reducing allostatic load in depression and anxiety disorders: physical activity and yoga practice as add-on therapies. Front Psychiatry.

[25] Zhang Y, Zhang X, Li J, Zhong H, Pan CW (2020). Associations of outdoor activity and screen time with adiposity: findings from rural Chinese adolescents with relatively low adiposity risks. BMC Public Health.

[26] Thompson Coon J, Boddy K, Stein K, Whear R, Barton J, Depledge MH (2011). Does participating in physical activity in outdoor natural environments have a greater effect on physical and mental wellbeing than physical activity indoors? A systematic review. Environ Sci Technol.

[27] Gorman S, Larcombe AN, Christian HE (2021). Exposomes and metabolic health through a physical activity lens: a narrative review. J Endocrinol.

[28] Mendoza MVF, Kachur SM, Lavie CJ (2022). The effects of exercise on lipid biomarkers. Methods Mol Biol.

[29] Joensuu L, Kujala UM, Kankaanpää A, Syväoja HJ, Kulmala J, Hakonen H, Oksanen H, Kallio J, Tammelin TH (2021). Physical fitness development in relation to changes in body composition and physical activity in adolescence. Scand J Med Sci Sports.

[30] Chung WK, De Vos-Jakobs S, Rivadeneira F, Bierma-Zeinstra SM, Waarsing JH (2021). The association of BMI and physical activity on acetabular dysplasia in children. Osteoarthr Cartil.

[31] Yu HJ, Li F, Hu YF, Li CF, Yuan S, Song Y, Zheng M, Gong J, He QQ (2020). Improving the metabolic and mental health of children with obesity: a school-based nutrition education and physical activity intervention in Wuhan, China. Nutrients..

[32] Contrepois K, Wu S, Moneghetti KJ, Hornburg D, Ahadi S, Tsai MS, Metwally AA, Wei E, Lee-McMullen B, Quijada JV, Chen S, Christle JW, Ellenberger M, Balliu B, Taylor S, Durrant MG, Knowles DA, Choudhry H, Ashland M, Bahmani A, Enslen B, Amsallem M, Kobayashi Y, Avina M, Perelman D, Schüssler-Fiorenza Rose SM, Zhou W, Ashley EA, Montgomery SB, Chaib H, Haddad F, Snyder MP (2020). Molecular choreography of acute exercise. Cell..

